# Processes of molecular adsorption and ordering enhanced by mechanical stimuli under high contact pressure

**DOI:** 10.1038/s41598-022-07854-5

**Published:** 2022-03-09

**Authors:** Seiya Watanabe, Chiharu Tadokoro, Koji Miyake, Shinya Sasaki, Ken Nakano

**Affiliations:** 1 Department of Mechanical Engineering, Tokyo University of Science, 6-3-1 Niijuku, Katsushika-ku, Tokyo, 125-8585 Japan; 2 Department of Mechanical Engineering, Saitama University, 255 Shimo-Okubo, Sakura-ku, Saitama, 338-8570 Japan; 3 Advanced Manufacturing Research Institute, National Institute of Advanced Industrial Science and Technology, 1-2-1 Namiki, Tsukuba, Ibaraki 305-8564 Japan; 4 Faculty of Environment and Information Sciences, Yokohama National University, 79-7 Tokiwadai, Hodogaya, Yokohama 240-8501 Japan

**Keywords:** Mechanical engineering, Techniques and instrumentation, Optical spectroscopy

## Abstract

Adsorbed molecular films, referred to as boundary films in tribology, are widely used in various industrial products as a keyway for surface functionalisation, such as lubricity, wettability, and adhesion. Because boundary films are thin nanometre-scale molecular layers and can easily be removed, their formation process cannot be elucidated in detail. In this study, to analyse the growth dynamics of boundary films, the film thickness and molecular orientation of the boundary film of a fatty acid used as an additive in rolling contact as mechanical stimuli were measured in situ. The measurements were performed on simple test lubricants, which were composed of n-hexadecane and stearic acid, at rolling tribological condition between steel and glass (or sapphire) surfaces by ultrathin film interferometry combined with sum-frequency generation spectroscopy according to a unique protocol. The results quantitatively demonstrate shear-induced boundary film formation. The insight gained from these results is anticipated to enable the formulation of high-performance lubricant additives to further reduce friction loss and high-performance glues that can be freely designed for removability.

## Introduction

Molecular adsorption is a fundamental phenomenon that is widely used as a basic technique to considerably modify material surface properties, such as wettability, molecular affinity, adhesion, and lubricity. The adsorbed films (e.g., self-assembled monolayers (SAMs)^1^) of only a few nanometres thick can afford practical surface properties for industrial, medical, and daily life applications. For example, when the film is used on only one surface, it can afford surface properties that are closely related to the hydrophobicity of liquid-repellent surfaces and adsorption sensitivity of biosensors. When used on two surfaces, the surface property is also closely associated with the adhesion of dissimilar materials and lubrication of mechanical systems.

The enhancement of hydrophobicity is induced by organosilane compound coating that is adsorbed on a glass surface. This technique is widely applied to produce rain-repellent vehicle windshields, self-cleaning building windows, and photovoltaic panels^2^. In their practical application, the liquid-repellent coatings must be nanoscale thick because transparency must also be achieved. Hydrophobicity, a property enhanced by suitable surface roughness, could also be achieved by molecular adsorption^3^. In biosensors, to detect the target analyte, molecular adsorption is used with high-sensitivity surface measurement, such as surface plasmon resonance (SPR) and quartz crystal microbalance (QCM)^4^. To improve biosensor sensitivity, the affinity with the target analyte is enhanced by surface modification using molecular adsorption methods, such as SAM^5^.

In terms of adhesion, adhesiveness and removability are important. For instance, with respect to biofouling and bio-adhesion of mussels, the interfacial hydration structure and protein between the surface and mussel adhesive plaque have been reported to perform an important role in antifouling. This means that materials that afford strong surface hydration exhibit excellent antifouling performance^6,7^. Bio-inspired adhesion has recently attracted considerable interest from another perspective. The mechanism of spider webs whose adhesion is resistant to interfacial failure under humid conditions has been investigated^8^. The study suggested that the hygroscopic components of the web sequester interfacial water and preserve the bond between the glue and surface. The responsiveness of the molecules adsorbed onto a surface to shear force accomplishes an important role in the removability performance. Previous studies have evaluated the surface properties of adsorbed films under equilibrium and static conditions. However, for improving performance in industrial applications, it is crucial to understand the responsiveness of these films to shear force.

In the field of tribology (i.e., the science and technology of friction, wear, and lubrication), the adsorbed molecular films (referred to as boundary films) are widely known to considerably affect the frictional and wear properties of associated materials. Substances comprising non-polar alkyl chains and polar groups, such as fatty acids, alcohols, and ethers, organic friction modifiers. For years, the mechanism of friction reduction with the application of organic friction modifiers has been discussed^9–12^. When added to the base oil at concentrations less than 0.1 wt%, the foregoing type of molecules covers the entire material surface and prevent direct contact between two sliding surfaces^13^. The adsorption characteristics of friction modifier molecules are described as follows. (1) The quantity of adsorbed molecules is governed by the functional groups of molecules and the adsorption sites on the surface. (2) Increasing the unsaturated bonds in organic friction modifiers leads to the formation of multiple layers by increasing the number of physisorbed molecules^14^. (3) The coverage of adsorbed unsaturated fatty acids compared with saturated fatty acids is lower because their structure is more disordered^15^. (4) At the initial stage of adsorption, island structures, which are domains of molecular adsorption film dots, are formed on the surface. Then, as the structures spread, the surface coverage area increases^16^.

In recent years, in situ measurements, as a means of investigating the practical role of organic friction modifiers under the dynamic phenomenon of friction, have attracted considerable attention. In this regard, a powerful tool for measuring the distance and interactive forces between sliding surfaces is a surface force apparatus (SFA). The measurements afforded by the SFA provide details of the friction mechanism, where confined and sliding structures are related to the presence or absence of organic friction modifiers. For example, it was demonstrated that the base oil on a surface coated with a single layer of organic friction modifier forms a molecular layer and behaves as a non-adsorbed layer, exhibiting solid-like slippage at the outermost surface of the friction modifiers layer^17^. SPR has also been used for the in situ measurement of the surface coverage of friction modifier molecules adsorbed onto gold surfaces in the presence of shear forces^18^. The results suggest that shear forces enhance the inflow of friction modifier molecules into the friction interface thus inducing adsorption. In situ measurements using infrared–visible sum-frequency generation (SFG) spectroscopy have also provided new insights into the molecular behaviour of base oil with friction modifier molecules at the friction interface under hydrodynamic lubrication, where two slid surfaces are separated by a thin film of a lubricant^19^. The adsorbed friction modifier molecules allow the base oil to orient along the sliding direction. This reduces fluid resistance by improving lubricant flow while the adsorption films behave as solid-like films. The alignment of liquid molecules, such as base oils near the surface induced by the alignment of surface molecules of the adsorption film, is similar to the liquid crystal alignment induced by rubbing polymer surfaces^20^.

Recently, the simulations of molecular dynamics (MD) have aided in understanding the relationship between the adsorption of friction modifier molecules and the alignment of base oil as well as that of friction modifier molecules^21,22^. The MD results suggest that the frictional property is not only governed by the nature of adsorbed additive films but also by the behaviour of the base oil molecules between two sliding surfaces. The hypotheses regarding the mechanism of frictional properties at the molecular level are expected to be experimentally verified by in situ measurements at the friction interface.

In terms of practical applications to mechanical components, such as ball bearings, the friction interface is subjected to high contact pressure and shear force exerted by the steel materials. However, under practical conditions, gaining insight on the behaviour of molecules remains exigent. This is because measuring the friction interface in situ is typically performed under restrictive conditions, such as under low contact pressure using alternative materials (e.g., mica, gold, and silica), which differ from those used in practice. Ultrathin film interferometry (UTFI) enables the in situ measurement of lubricant film thickness to less than 5 nm at the friction interface under high contact pressure using a pair of steel ball and glass plate^23^. Through UTFI, the static contact in a solution comprising a lubricant with a friction modifier was found insufficient to initiate boundary film formation. Under high pressure, the rolling contact acts as a trigger for the formation of this boundary film to separate the points of contact^24^. The use of an empirical approach to analyse when and how an adsorbed film containing additives is formed on the surface is important for the development of lubricating oils that maximise additive performance.

In this study, we focused on the effect of rolling motion as a mechanical stimulus on the formation of an adsorbed layer of a simple model lubricant, specifically stearic acid (SA) dissolved in n-hexadecane (HD). The UTFI and SFG spectroscopy analyses were conducted using the same tribo-rig and motion protocol to explain the cause and effect of changes in the film thickness and the orientation of molecules in the contact on the formation of adsorbed film.

## Results

### Film thickness measurements

**Figure 1:**
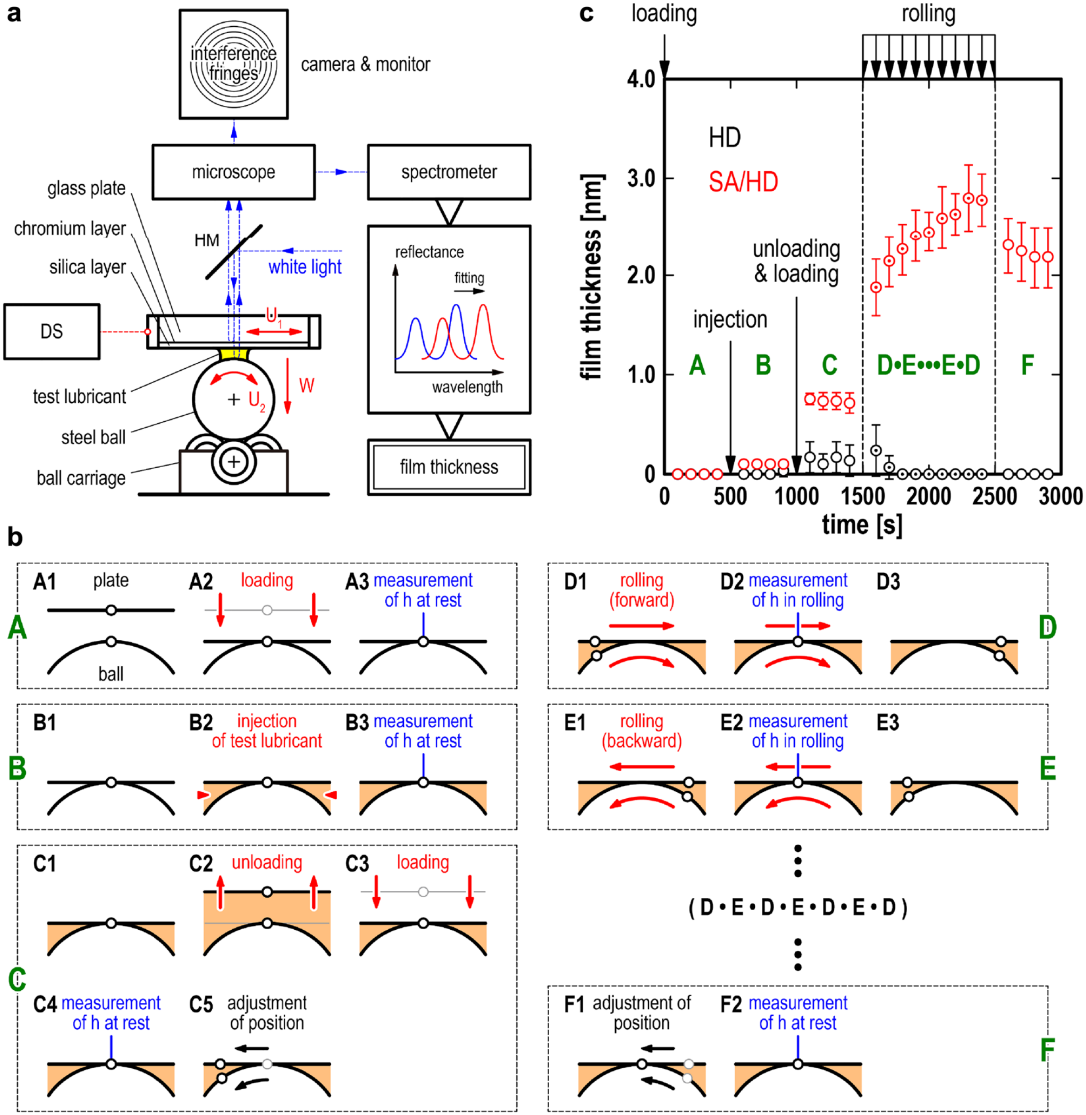
Film thickness measurements. (**a**) Schematic of the custom-built UTFI apparatus, which consisted of spectroscopy measurement system and pure rolling reciprocating rig for measuring the film thickness of the test lubricant under pure-rolling contact (*U*_1_ = *U*_2_); DS: displacement sensor and HM: half mirror; (**b**) Experimental protocol—A: after loading from the non-contact state without any lubricants, the film thickness was measured under static contact conditions; B: after injecting a test lubricant into the contact point, the film thickness was measured under static contact conditions; C: after unloading and reloading, the film thickness was measured under static contact conditions, and then the plate and ball were moved with the pure rolling motion for half a stroke back from the origin; D: during the pure rolling motion for a forward stroke, the film thickness was measured at the origin; E: during the pure rolling motion for a backward stroke, the film thickness was measured at the origin; F: after the plate and ball were moved with the pure rolling motion for half a stroke back to the origin, the film thickness was measured static contact; (**c**) Temporal changes in the film thickness of the HD and SA/HD lubricant films according to the protocol; black plots and error bars: the mean and standard deviation of the three-time measurements of the HD; red plots and error bars: the mean and standard deviation of the five-time measurements of the SA/HD.

Film thickness measurements were performed using a custom-built UTFI apparatus, consisting of a spectroscopy measurement system and a tribo-rig with a point contact between a steel ball and a glass plate, as shown in Fig. 1a. The glass plate, coated with approximately 10 and 600 nm of chromium and silica layers as semi-reflection and spacer layers, respectively, was used as specimen to generate high-contrast interference at the contact. The lubricant film thickness between the two surfaces at the contact was measured using the UTFI. To avoid the influence of measurement position, (e.g., variations in the thickness of chromium layer or silica layer and surface roughness), the measurement was conducted according to the following protocol (Fig. 1b). The experimental protocol consists of six steps (A–F), which are explained in detail in the Methods section. In step A, after loading from the non-contact state without lubricants, the film thickness was measured under a static contact condition. In step B, after injecting a test lubricant, the film thickness was measured under a static contact condition. In step C, after unloading and reloading, the film thickness was measured under a static contact condition; then, the plate and ball were moved from the origin, which was the position in steps A–C, by pure rolling for half a backward stroke. In step D, the film thickness was measured at the origin during the pure rolling motion for a forward stroke. In step E, the film thickness was measured at the origin during the pure rolling motion for a backward stroke. Steps D and E were alternately repeated nine times. In step F, the film thickness was measured under a static contact condition after removing the plate and ball by pure rolling motion to the origin for half a backward stroke. The difference in the film thickness among the processes clearly shows the effect of motion in each step on film formation.

The film thickness measurements according to the above protocol were conducted on HD and HD with SA (SA/HD) lubricant films, as shown in Fig. 1c. The continuous measurement conducted at the origin according to the protocol can precisely measure the film thickness to a resolution of 0.1 nm (Fig. S1, Supplementary Information). In step A in Fig. 1c, the film thickness (*h*) of both test lubricants was 0 nm at *t* = 100–400 s because the contact point was under a dry static condition. In step B, 100 mm^3^ of the test lubricant was injected into the contact point at *t* = 500 s, causing a slight change in the *h* value of SA/HD during *t* = 600–900 s. By contrast, for HD, the test lubricant injection did not affect the film formation during *t* = 600–900 s. In step C, the contact point was unloaded to separate the surfaces and then reloaded at *t* = 1000 s, generating a lubricant film of *h* = 0.1–0.2 nm for HD; in addition, *h* = 0.6–0.7 nm for SA/HD during *t* = 1100–1400 s. In steps D and E, for HD, the lubricant film was eliminated by the rolling contact process. In contrast, for SA/HD, the first rolling contact at *t* = 1600 s formed the lubricant film with *h* = 1.9 nm, and the film was definitely developed after eight subsequent rolling contacts, reaching *h* = 2.8 nm at *t* = 2400 s. In step F, after the rolling contact process, the lubricant film of SA/HD under the static contact condition during *t* = 2600–2900 s was *h* = 2.2 nm, which was thicker than that during *t* = 1100–1400 s. The 0.6-nm difference between the lubricant film thickness after the rolling contact process at *t* = 1600 s and after the static contact process during *t* = 2600–2900 s indicates the fluid film thickness. This is because the film thickness reduction indicates that the fluid film was forced out of the contact point. In other words, the lubricant film with *h* = 2.8 nm at *t* = 2400 s consisted of the adsorbed and fluid films. These results indicate that the adsorbed film was formed in two steps: rapid growth by the first rolling contact and slow growth by subsequent rolling contacts.

### SFG measurements

**Figure 2:**
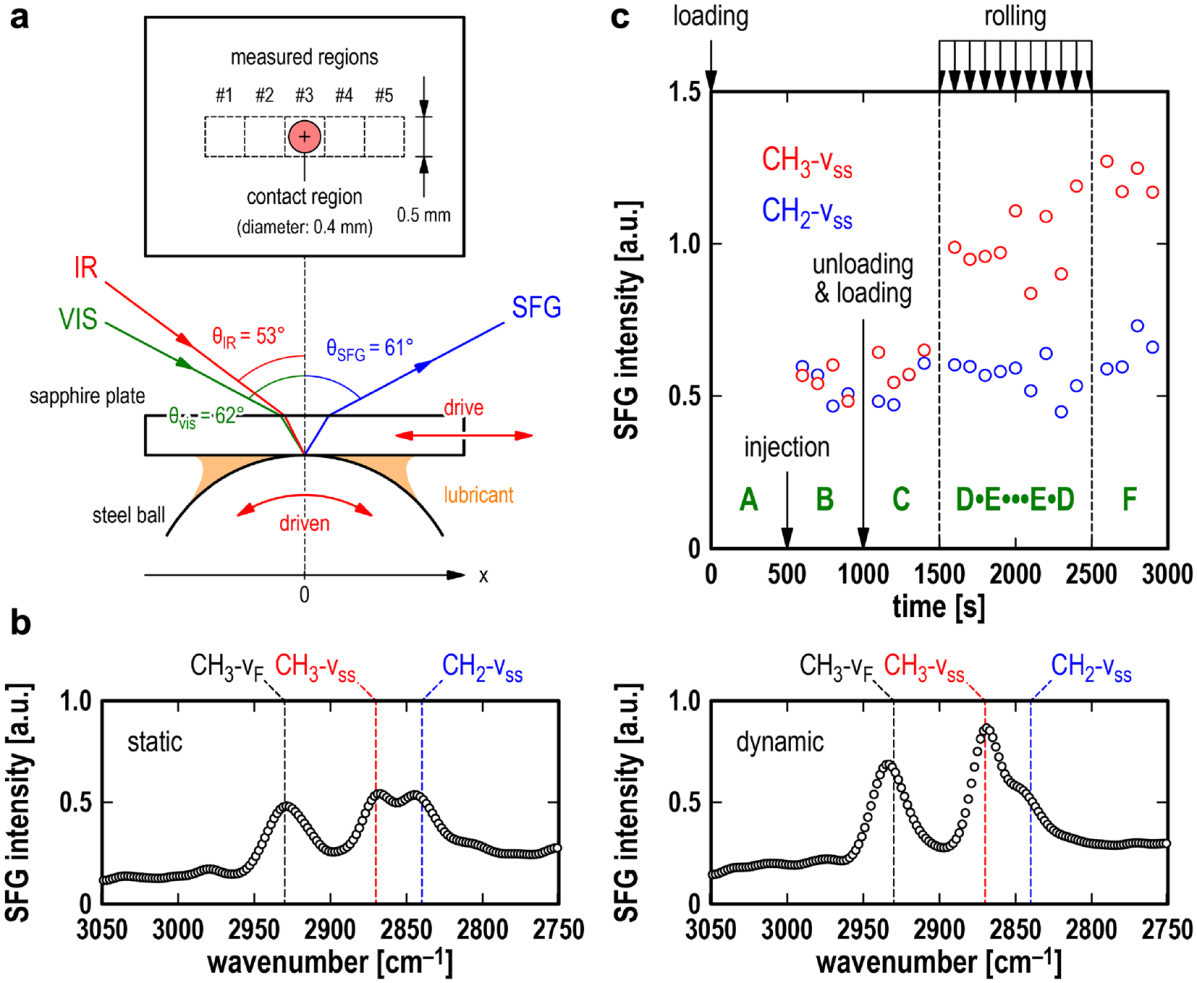
SFG measurements. (**a**) Schematic of the SFG apparatus using the pure rolling reciprocating rig, which is the same as the UTFI apparatus. (**b**) SFG spectra of SA/HD-d_34_ under static and dynamic conditions. (**c**) Temporal change in the intensity of CH_2_-vss and CH_3_-vss of SA/HD-d_34_ measured according to the same rig motion protocol as the film thickness measurements.

First, the SFG spectra under the static and dynamic (successive reciprocating rolling) contact conditions were acquired (Fig. 2a) to understand the differences in the intensities and positions of the SFG peaks between the two conditions. The SFG spectrum of deuterated HD with SA (SA/HD-d_34_) (Fig. 2b) in the wavelength range of the C–H stretching mode reflected the molecular behaviour of the adsorbed SA film. The peaks observed at 2840, 2870, and 2930 cm^−1^ were attributed to the CH_2_ symmetric stretching mode (CH_2_-v_ss_), CH_3_ symmetric stretching mode (CH_3_-v_ss_), and CH_3_ Fermi resonance (CH_3_-v_F_), respectively. The intensities of the CH_3_-v_ss_ and CH_3_-v_F_ peaks were higher under rolling contact conditions than those under static contact conditions. By contrast, upon focusing on HD (SFG spectra of SA-d_35_/HD shown in Fig. S2, Supplementary Information), the HD molecules clearly do not differ under the static and dynamic contact conditions. Subsequently, an experiment following the same protocol as the film thickness measurement was performed on SA/HD-d_34_; the time-dependent changes in the SFG intensities were monitored. Figure 2c shows the measurement results as a function of time. The change in the CH_2_-v_ss_ intensity was slight throughout the entire test sequence (*t* = 600–2900 s). The CH_3_-v_ss_ peak showed a small change in intensity before the rolling motion started (*t* = 600–1400 s). When the contact condition changed from static to rolling (*t* = 1600 s), the intensity of the CH_3_-v_ss_ peak increased by 1.67 times. Thereafter, the intensity increased with the number of rolling motions (*t* = 1600–2400 s) and oscillated after the fifth rolling step (*t* = 2000–2400 s). Under the static contact condition after the rolling motion (*t* = 2600–2900 s), the intensity exhibited a negligible change from the intensity at the end of the rolling process (*t* = 2400 s). The tendency of the CH_3_-v_ss_ intensity to increase and oscillate was analysed in terms of the changes in the tilt (*θ*) and azimuthal (*χ*) angles of the CH_3_ groups. The analysis is explained in detail in the Supplementary Information (*Orientation analysis of the CH*_*3*_* group*) section. The results indicate that the CH_3_ end-groups, then SA, became oriented along the rolling motion direction after the first rolling process. As the number of rolling processes increased, they tended to become perpendicularly oriented to the surface.

## Discussion

This section discusses the interfacial phenomenon observed in the experiments for each step of the protocol (Steps A–F) according to the experimental results of film thickness and orientation of molecules obtained from both film thickness and SFG measurements, respectively. In step B, in the case of HD without SA, after the test lubricant injection (*t* = 600–900 s), the film thickness was 0 nm (Fig. 1c), and no SFG peaks were observed (Fig. S3, Supplementary Information). These results indicate that the HD molecules do not have a regularly ordered structure, or they do not exist between the surfaces. By contrast, for the SA/HD case during the same period (Fig. 1c), the film thickness became 0.1 nm, and peaks corresponding to the adsorbed film for SA/HD-d_34_ were observed by SFG (Fig. 2c). Note that the film thickness measured by the UTFI is spatial average value as described in the Methods (*Film thickness measurements*) section. These results indicate that SA can penetrate partly the contact point just by injecting a lubricant because the results of the UTFI do not necessarily imply that a 0.1-nm uniform layer was generated. In step C, for the SA/HD case, after unloading and loading (*t* = 1100–1400 s), the film thickness increased to 0.5 nm. The structure of the SA-adsorbed film did not change because there was no change in the SFG spectrum. These results indicate that because of the mechanical action of unloading and loading, the adsorption of both SA and HD was induced by exposing the surfaces to the lubricant. In step D, at the first rolling step (t = 1600 s), the film thickness (Fig. 1c) and intensity of the CH_3_-vss peak (Fig. 2b) increased after the rolling motion. The film thickness measurement results indicated that the formation of the lubricant film was stimulated by the lubricant’s penetration of the interface because of the rolling motion. The lubricant film thickness was approximately 1.9 nm, which was smaller than the size of SA in the all-trans configuration (2.4 nm). The detailed analysis results of the SFG show that the CH_2_-vss peak intensity remained unchanged before and after the first rolling motion. The analysis of the intensity ratio of the CH_3_-vss peak under static and dynamic conditions (Fig. S4, Supplementary Information) indicated that the tilt angle of the CH_3_ end group was approximately 90°, which was defined as parallel to the surface. In addition, the azimuthal angle of *χ* indicates that the CH_3_ end-group is aligned parallel to the rolling direction. Accordingly, based on these results, the boundary film composed of adsorbed SA was formed at the first rolling motion; however, its density was not high. Therefore, the adsorbed SA was likely to tilt along the rolling direction, resulting in a lubricant film thickness smaller than the SA size in the all-trans configuration. The lubricant film thickness gradually increased with the number of reciprocating rolling motions. After nine reciprocating rolling motions (t = 1700–2400 s), the lubricant film thickness reached 2.8 nm. Furthermore, a detailed analysis of the SFG spectra indicated that the tilt angle of the CH_3_ end group slightly decreased in increments of 0.5° as the number of reciprocating rolling motions increased. Previous molecular-dynamics simulations studying adsorption film of stearic acid on iron oxide indicated that the surfactant molecules point upwards at high surface coverage^25^. Based on this, we believe that the increase in the density of adsorbed SA molecules led to a decrease in the tilt angle and an increase in the lubricant film thickness. These results are consistent with the previous SPR^18^ and UTFI^24^ results, that is, the rolling contact acts as a trigger for boundary film formation, separating the solid surfaces at the contact point under high pressure. The SFG results further suggested that forward and backward rolling motions induced the CH_3_ terminal group to swing back and forth during the latter part of the reciprocating rolling motion (t = 2000–2400 s). This may be the result of the coordinated behaviour among densely adsorbed SA molecules combined with the reciprocating rolling motion. After the rolling motion halted (t = 2600–2900 s), the lubricant film thickness decreased to 2.2 nm; however, the SFG spectra exhibited a slight change. These results imply that the lubricant film, which was measured during the rolling motions in steps D and E, was composed of boundary and fluid films corresponding to the adsorbed SA film and fluid HD, respectively. The fluid HD was discharged from the contact point, leading to a decrease in the film thickness after the rolling motion stopped. The boundary film of the adsorbed SA exhibited a slight change.

**Figure 3:**
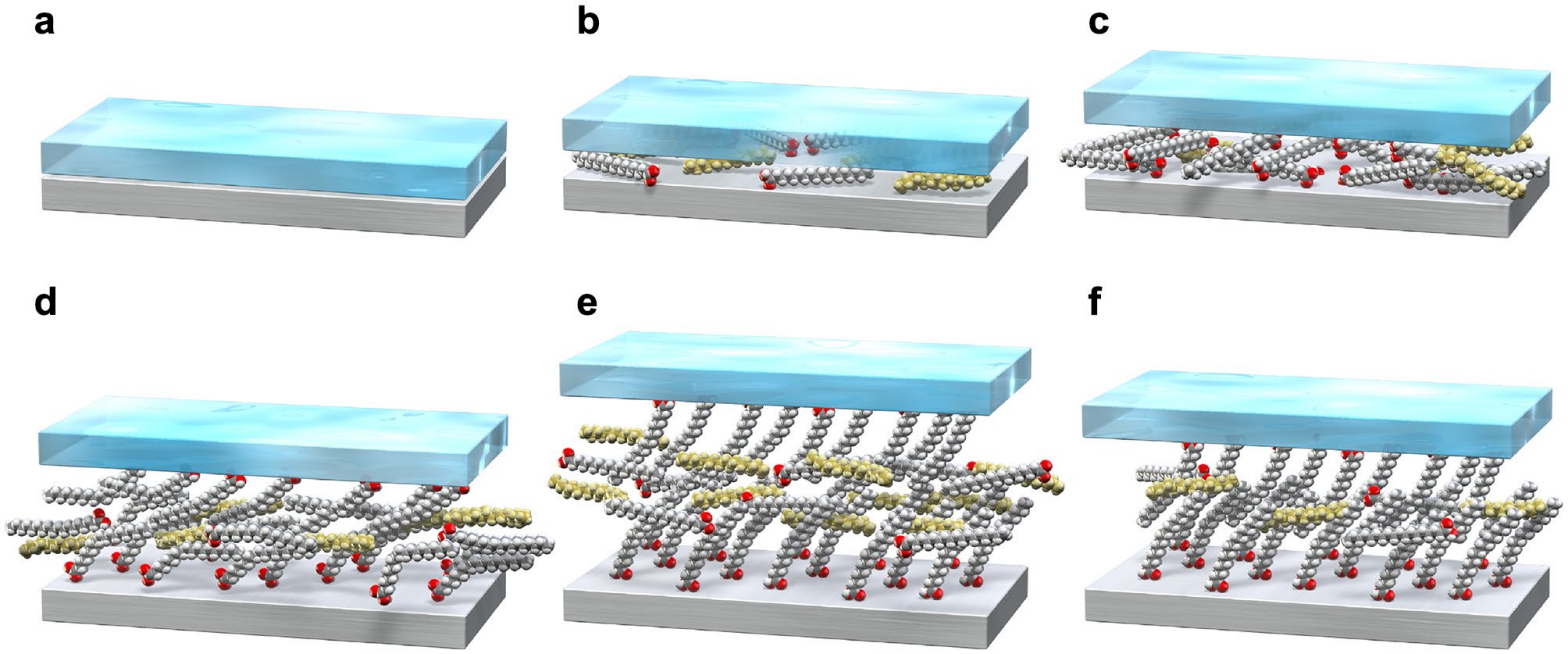
Schematic representation of the boundary film formation process; white molecules: SA; yellow molecules: HD; red atoms: oxygen. (**a**) Static contact before lubricant addition. (**b**) Penetration of lubricant into static contact. (**c**) Enhanced adsorption of SA by unloading and loading. (**d**) Boundary film formation of SA by applying first rolling contact shear force; driving direction of upper plate: right-hand side. (**e**) Growth and ordering of SA film by subsequent rolling contacts; driving direction of upper plate: right-hand side. (**f**) Residual boundary film of SA at static contact after rolling contact.

Figure 3 shows a schematic of the model of the boundary film formation process based on the UTFI and SFG spectroscopy results. Initially, SA and HD penetrated the contact point upon the injection of the lubricant alone (Fig. 3a, b). Then, the penetration of the lubricant upon unloading and loading enhanced the adsorption of SA, increasing the lubricant film thickness (Fig. 3c). The first rolling motion induced the formation of a boundary film composed of adsorbed SA; however, its molecular order is not high owing to the low density (Fig. 3d). The lubricant film thickness and molecular order and density of adsorbed SA gradually increased with the number of reciprocating rolling motions (Fig. 3d, e). After the rolling motions, under the static condition, the fluid film was forced out of the contact point, but the boundary film remained at the contact point, while maintaining the order (Fig. 3f). These indicate that the shear force acting on the interface performs an important role in the formation of the boundary film, that is, the shear force draws the lubricant into the friction interface and enhances the adsorption and ordering of additive molecules on the surface.

This study demonstrates that measurements combining in situ UTFI and SFG spectroscopy according to the unique protocol provide insight into the process of adsorbed film formation under rolling contact conditions. The foregoing approach affords three advantages: (1) the measurement results of the UTFI and SFG spectroscopy complement one another; (2) the technique can be applied to the examination of transient phenomena; and (3) the protocol, which can be continuously implemented for measurements under various conditions, enables the comparative analysis of each situation. Previous detailed investigations also were conducted by considering the combination of measurement methods. Chen et al.^26^ examined the process of adsorption or self-assembly of surfactant monolayers and bilayers from a solution onto mica surfaces using X-ray photoelectron spectroscopy (XPS) and SFA. The combination of XPS and SFA provided insights based on physical and chemical information. Ralf et al.^27^ examined the mechanism of supported lipid bilayer formation and vesicle adsorption on mica surfaces using QCM with dissipation (QCM-D), atomic force microscopy (AFM), and ellipsometry. The measurement methods in this combination complement each other in terms of affording temporal and spatial information for quantitative comparison. By contrast, Gosvami et al.^28^ examined the mechanism of anti-wear tribofilm growth using AFM from the perspective of continuous transient phenomenon measurement. Continuous measurement is effective for understanding the effects of mechanical stimuli on transient phenomena. Previous studies clearly show that continuous measurement enables the formulation of a consistent model by capturing a phenomenon from different angles using multiple metrics, thus elucidating the temporal changes in the phenomenon. Dynamic phenomena, such as molecular adsorption and tribology, can only be accurately explained by the combination of multiple and continuous measurement methods. In this study, an investigation that combined continuous in situ measurements using UTFI and SFG spectroscopy was conducted, gaining novel insights into the process of adsorbed film formation. The combination of continuous in situ measurements, particularly those that are defined by measurement protocols under various conditions similar to this study, can afford further understanding of the effects induced by mechanical stimuli. To obtain further details of the phenomenon and distinguish between physical and chemical adsorption^29,30^, expanding the measurement wavenumber range of SFG spectroscopy to the C=O stretching mode region is effective; however, the window material has to be thoroughly considered. In addition, to discuss the island structures of adsorbed films^16^, developing a UTFI for measuring the film thickness distribution is preferred^31^.

In summary, this paper proposes a model for boundary film formation based on the adsorption behaviour of SA dissolved in HD and deduced from film thickness and SFG measurements. The findings are widely applicable to techniques that utilise molecular adsorption phenomena, such as hydrophobicity for liquid-repellent surfaces, adsorption sensitivity of biosensors, adhesion control as well as removability, and lubrication of mechanical systems. In particular, the proposed approach affords gaining insight into the friction reduction mechanism of organic friction modifiers leading to the development of high-performance lubricant additives. The technique developed in this study aids in understanding the responsiveness of adsorption molecules to shear forces. Furthermore, the responsiveness of adsorption molecules against shear forces is related to adhesiveness and removability of glues. Accordingly, such understanding can contribute to the development of antifouling coatings and biomimetic adhesive materials. The reported results and method are anticipated to considerably affect future research in chemistry, nanoscience, and nanotechnology, because the in-depth understanding of interfacial molecular dynamics and lubrication mechanisms is essential to these fields.

## Methods

### Test lubricants

In the experiment, HD (> 99% pure) and SA (free acid, > 99% pure), which were from Wako Pure Chemical Industries Ltd. (Osaka, Japan), were used as base lubricant and organic friction modifier, respectively. Moreover, to distinguish the C–H stretching mode peaks of SA from those of HD in the SFG measurements, HD-d_34_ (99.3% rate of deuteration), which was from Kanto Reagent (Tokyo, Japan), was used as another base lubricant. The organic friction modifier was dissolved in the base lubricant at a concentration of 3 mM (approximately 0.1 mass%).

### Film thickness measurements

The film thickness measurements were performed using a custom-built UTFI apparatus. As shown in Fig. 1a, the apparatus is equipped with a spectroscopic measurement system and a pure rolling reciprocating rig with a point contact between a steel ball (material: AISI 52100 steel; diameter: 19.1 mm; arithmetic average roughness: 16 nm) and a glass plate (material: borosilicate glass; diameter: 50 mm; thickness: 5 mm; arithmetic average roughness: 4 nm). The normal load of the point contact was controlled by a motorised linear stage and lever mechanism. The contact diameter and the mean contact pressure were estimated to be 260 µm and 0.4 GPa, respectively, at the normal load of 20 N by the Hertz contact theory. The steel ball was placed on a ball carriage consisting of three inclined ball bearings that allowed the steel ball to rotate freely with minimal friction. A motorised stage drove the glass plate linearly in a reciprocating manner, while a displacement sensor monitored the glass plate. The linear motion of the glass plate drove the steel ball in a nominally pure rolling contact. The glass plate surface in contact with the steel ball was coated with a semi-reflecting layer of chromium (approximately 10 nm thick) and a spacer layer of silica (approximately 600 nm thick) over the chromium. The white light reflected by a half mirror passed through an objective lens (magnification: 20×) and illuminated the contact point. A certain amount of this light was reflected by the chromium layer, whereas some other amounts passed through the spacer layer and lubricant film and then reflected by the steel ball. To derive the reflectance spectrum, the interfered light with a diameter of 20 µm (spot size for the UTIF) at the contact centre extracted through a 20× magnification objective lens and a 400-µm diameter glass fibre was directed to a spectrometer. The exposure time of the spectrometer was 2 ms. A computer program SCOUT, which is a thin film analysis software, determined the film thickness of the test lubricant; consequently, the theoretical reflectance spectrum in an optical model of the contact point corresponded to that obtained experimentally. A displacement sensor monitored the location of the glass plate during the rolling contact. During the rolling contact, the film thickness of the test lubricant was analysed through the automatically measured reflectance spectrum at the origin of the glass plate. The origin was detected by a signal from the displacement sensor, which avoided possible errors caused by the thickness and microtopography of the spacer and semi-reflecting layers.

The experimental procedure for measuring the film thickness proceeded as follows. First, the steel ball, glass plate, and ball carriage were cleaned in acetone and in hexane using ultrasonic cleaner, dried in a hot air stream, and set in the rig. Second, the thickness values of the semi-reflecting and spacer layers were determined under a normal load of 20 N. Then, a series of film thickness measurements were performed according to the protocol (Fig. 1b), as elaborated below. In step A, (A1) from the non-contact state without any lubricants, (A2) a load was placed on the glass plate such that the normal load between the steel ball and the glass plate was *W* = 20 N at *t* = 0 s. (A3) The film thickness was measured under a static contact condition every 100 s during *t* = 100–400 s. In step B, (B1) from the static contact state without any lubricants, (B2) at *t* = 500 s, 100 mm^3^ of the test lubricant was injected around the contact point using a syringe while maintaining *W* = 20 N. (B3) The film thickness was measured under a static contact condition every 100 s during *t* = 600–900 s. In step C, (C1) from the static contact state lubricated by the test lubricant, (C2) the contact point was unloaded at *t* = 1000 s to separate the surfaces and then (C3) reloaded at *W* = 20 N. (C4) The film thickness was measured under a static contact condition every 100 s during *t* = 1100–1400 s. (C5) At *t* = 1500 s, the glass plate and steel ball were moved backwards by half of the stroke (i.e., *L*/2 = 0.5 mm) from the origin with pure rolling motion at *U*_1_ = *U*_2_ = 0.1 mm/s, where *U*_1_ and *U*_2_ are the plate translational and ball rotational velocities, respectively. In steps D and E, the glass plate and steel ball were moved forwards and backwards, respectively, by the stroke *L* = 1.0 mm at *U*_1_ = *U*_2_ = 0.1 mm/s. During the pure rolling motion, the spectrum for calculating the film thickness was measured at the origin using the displacement sensor as a trigger. Steps D and E were alternately repeated nine times every 100 s during *t* = 1600–2400 s. This means that there was a 90 s interval between pure rolling motions of the steps. In step F, (F1) at *t* = 2500 s, the plate and ball were moved with pure rolling motion for half a stroke (*L*/2 = 0.5 mm) back to the origin at *U*_1_ = *U*_2_ = 0.1 mm/s. (F2) The film thickness was measured under a static contact condition every 100 s during *t* = 2600–2900 s. The difference in the film thickness among the steps clearly demonstrated the effects of the action in each step on the film formation. The ambient temperature and relative humidity during this procedure were 25 °C and 20%–40%, respectively.

### SFG measurements

The SFG measurements were performed using a pure rolling reciprocating rig with a point contact between a steel ball and a sapphire (Al_2_O_3_) plate. The SFG apparatus is shown in Fig. 2a. The apparatus used a mode-locked Nd:YAG laser generating a 1064-nm fundamental pulse beam with a width and frequency of 30 ps and 10 Hz, respectively. Fixed visible and tuneable infrared beams (532 nm and 2.5–10 μm, respectively) were generated by an optical parametric generator/optical parametric amplifier system. The visible and infrared beams overlapped at the sample surface with incident angles of 62° and 53° relative to the surface normal, respectively. The representative input energy levels of the visible and infrared beams were set to ~ 50 and ~ 250 μJ/pulse, respectively. The polarisation combination of measurements was ssp (SFG, visible, infrared). Here, s and p represent perpendicular and parallel polarisations, respectively. The SFG spectra of HD-d_34_/SA were obtained under static and rolling conditions with a load of 20 N. The diameter of the laser spot on the surface of visible (VIS) and infrared (IR) light were approximately ϕ 400 μm and ~ ϕ 150 μm, respectively. The contact diameter deduced by the Hertz contact theory was also approximately ϕ 200 μm. In order to confirm that the overlapped laser spots of the VIS and IR light within the contact interface, the position dependence of the SFG spectra was measured (Fig. S6, Supplementary Information). The result indicates that the detected SFG signal was generated within the contact region of the Al_2_O_3_ plate and AISI 52100 ball surfaces. Two types of SFG measurements were performed to observe the SA behaviour. The first is (a) a typical measurement in the wavenumber range of 2750–3050 cm^−1^ where C–H stretching vibrational modes appear. The second is (b) a time-dependent measurement with the same protocol as that of the film thickness measurement at wavenumbers of 2840 and 2870 cm^–1^ corresponding to the peak positions of CH_2_-vss and CH_3_-vss, respectively. In the time-dependent measurement, to achieve an adequate time resolution, the SFG measurements were conducted with two selected wavenumbers. The number of SFG datasets was set at 10, and the data at selected wavenumbers were acquired alternately every 13 s. The plot in Fig. 2c shows the average data for 100 s.

## Supplementary Information

Supplementary Information.
