# Supplementary material for: Processes of molecular adsorption and ordering enhanced by mechanical stimuli under high contact pressure
Source: Sci Rep. 2022 Mar 9;12:3870. doi: 10.1038/s41598-022-07854-5 (PMC8907199; doi:10.1038/s41598-022-07854-5)
Supplement: Supplementary file 1 — Supplementary Information. [file 41598_2022_7854_MOESM1_ESM.docx]

**Supplementary Information**

*1. Resolution of film thickness measurement*

Figure S1 shows the typical reflectance spectra results obtained experimentally to determine the film thickness of the lubricant measured by UTFI. The reflectance spectra were calculated with the spectroscopic measurement system as described in the Methods (*Film thickness measurements*) section using the reference relationship between the known reflectance spectrum and measured intensity spectrum of the silicon wafer. The film thickness of the lubricant was determined by fitting the numerical reflectance spectrum to the experimental reflectance spectrum using a computer program SCOUT, which is a thin film analysis software; the reflectance spectrum was in the wavelength range 450–700 nm. Figure S1 shows a peak in the reflectance spectrum in the range 570–585 nm that is a third-order fringe in the optical interference. The wavelength resolution in the reflectance spectrum was 0.35 nm. The figure further shows the distinct differences in the reflectance spectrum between the lubricated (at *t* = 900, 1400, and 1600 s) and dry (at *t* = 100 s) conditions. The difference of the peak wavelength in the third-order fringe between the lubricated and dry conditions shows approximately the film thickness of lubricant. The equation of the relationship between the film thickness of lubricant *h* and the difference of the peak wavelengths *λ*_1_, *λ*_2_ is as follows:

$h=\left( \lambda_{2}-\lambda_{1} \right)\frac{N}{2n}$ (1)

where *N* and *n* are the fringe order (*N* = 3) and the refractive index (*n* ≈ 1.4). In Fig. S1, the solid curves represent fitting curves for each spectrum by the second-order approximation. After the lubricant was injected into the contact point (at *t* = 900 s), the peak value of the reflectance decreased; however, the peak wavelength remained clearly unchanged. Table S1 shows the influence of the centre wavelength of the second-order approximation on the coefficient of determination (*R*^2^). At *t* = 100 s, the peak wavelength *λ*_1_ is 576.0 nm because the centre wavelength of 576.0 nm is the minimum in *R*^2^. At *t* = 900 s, the peak wavelength *λ*_2_ is 576.1 nm because the centre wavelength of 576.1 nm is the minimum in *R*^2^. Therefore, it is confirmed that the film thickness of lubricant was 0.1 nm at *t* = 900 s. By contrast, after the normal load was reapplied (e.g., *t* = 1400 s), the increase in the film thickness (0.7 nm) evidently modified the wavelength. This result shows that a 0.1-nm-scale change in the film thickness can be detected by the film thickness measurement technique. During the first rolling contact (at *t* = 1600 s), the 2.0-nm film thickness was sufficiently large to be measured because the variation in the film thickness caused a significant change in the wavelength of the reflectance spectrum. This high resolution could be achieved by measuring the film thickness at the same position during the experimental protocol, as shown in Fig. 1(b).

*2. SFG spectrum of SA-d_35_/HD*

To understand the molecular behaviour of HD in the SA/HD solution, SA-d_35_ (with a 99.3% rate of deuteration) was used to distinguish the molecular information from the HD and SA. The SFG measurements under the same conditions as those in Fig. 2(b) are performed using SA-d_35_/HD; the results are shown in Fig. S2. Peaks were observed at 2840, 2870, and 2940 cm^−1^ and attributed to CH_2_-v_ss_, CH_3_-v_ss_, and CH_3_-v_F_, respectively. A negligible change under static and rolling contact conditions was observed, indicating that the HD molecules between the adsorbed SA films existed according to some order. Nevertheless, their structure under static and dynamic conditions remained unchanged.

*3. SFG spectrum of HD*

Figure S3 shows the SFG spectra of HD under static and rolling contact conditions. No peak was detected, suggesting two possible scenarios: (a) HD did not exist at the friction interface, or (b) HD existed with a completely random orientation.

*4. Orientation analysis of CH_3_ group*

Figure S4 shows the experimental fitting results for these parameters. Herein, we explain the overview of the orientation analysis of CH_3_ group by SFG measurement.

In this measurement, the SA films adsorbed on the Al_2_O_3_ plate and steel ball were detected. To determine the surfaces on which SA preferentially adsorbed, QCM measurements of the HD/SA were performed against Al_2_O_3_ and steel surfaces. Figure S5 shows the area density of the adsorbed film calculated from the *Δf* value obtained from the QCM measurements. Based on the results, the difference between the area densities of the SA films adsorbed on the Al_2_O_3_ and steel surfaces was inconsequential. This suggested that the adsorbed SA molecules were oppositely oriented in the vertical and in-plane directions, leading to the cancellation of a portion of the SFG signals. However, the SFG signals were detected in the experiments as reported in the manuscript. The foregoing can be explained as follows. The difference between the area densities of the adsorbed SA films on the Al_2_O_3_ plate and steel ball was negligible. The opposite orientation of the SA films cancelled the SFG signal, and the consequent balance of the SFG signals was detected. Based on the above hypothesis, the molecular orientation analysis of the methyl groups of the adsorbed SA film under static and dynamic conditions was performed.

As shown in Fig. 2(c), the difference in the CH_2_-v_ss_ intensity under the static and rolling contact conditions was negligible, indicating that there was no significant change in the area density of the adsorbed SA films when the contact condition changed from static to rolling. In contrast, the intensity of the CH_3_-v_ss_ peak suddenly increased after the transition from the static to the rolling contact condition. This indicated that the isotropic orientation of the CH_3_ groups in the in-plane direction decreased. Accordingly, the azimuthal orientation component, *χ*_yyx_, was added to the SFG intensity.

The intensity oscillation was observed at *t* = 2000–2400 s. The orientation angle analysis indicated that the intensity varied when the azimuthal angle orientation changed from 0° to 180° (the direction parallel to the incident plane of the light was defined as 0°). Assuming that the CH_3_ groups were oriented along the rolling direction, the intensity changes in the experimental results reflected a change in the azimuthal orientation between the forward and backward directions. However, the intensity oscillation amplitude at the start of the rolling contact was relatively weak. Hence, with the increase in the number of rolling steps, the in-plane orientation of the CH_3_ groups tended to gradually align with the frictional direction.

The change in the orientation angle of the CH_3_ group and its distribution were analysed based on the foregoing discussion. The focus of the analysis was on the CH_3_ intensity behaviour that suddenly increased at *t* = 1600 s, continued to increase during *t* =1600–2400 s, and oscillated during *t* =2000–2400 s. Wei et al. performed orientational analysis on a rubbed 8CB crystal adsorption film and obtained satisfactory fitting results with the assumption that the orientational distribution was Gaussian^[1]^. Here, the distribution of the azimuthal orientation angle was assumed to be Gaussian, with a median at the rolling direction angle (0° and 180°). The data plot of CH_3_-vss was fitted with the following five parameters: (a) tilt angle under a static condition (*θ*_S_), (b) tilt angle under a rolling contact condition (*θ*_D_), (c) change in the tilt angle by *Δθ*_D_ after every rolling step (a decreasing change in *θ*_D_ was positive), (d) standard deviation in the azimuthal angle (*σ*), and (e) change in the standard deviation (*Δσ*) after every rolling step (a decrease in *σ* was positive). The specific formulae are explained in the next section. The fitting parameters satisfying the experimental data are listed in Table S2. The tilt angle under the static contact condition (*θ*_S_) had a strong correlation with the standard deviation of the azimuthal angle (*σ*); *θ*_S_ was 37°–80°, and *σ* was 5°–125°. When rolling started, the tilt angle, *θ*_D_, under the rolling contact condition was 90°. Then, the tilt angle and standard deviation of the azimuthal direction changed with increasing rolling number; *Δθ*_D_ and *Δσ* became 0.1–0.5 and 0.2, respectively.

The results summarised in Table S2 clearly indicate that the fitting results considerably depend on the tilt angle, *θ*_S_, under the static condition. Based on the QCM measurements, the occupied area per molecule of the adsorbed film was calculated to be approximately 50 nm^2^ for the Al_2_O_3_ and steel surfaces. Therefore, the adsorbed SA film was considerably coarse and had a large tilt angle (*θ*_S_) distribution. A random orientation between 0 and 180° will lead to SFG signal with polarisation dependency corresponding to the so-called “magic angle” ^[2, 3]^. As for the random orientation of the adsorbed film, the tilt angle (*θ*) converged to the magic angle (*θ* = 39.2°) In the measurement and analysis herein, the dispersion of the tilt angle, *θ*_S_, under the static condition was hypothesised to be large; consequently, the tilt angle (*θ*_S_) obtained a value of approximately 39.2°. To calculate the intensity ratio of CH_3_-ss to CH_3_-as, the intensity levels of both peaks are necessary. However, the CH_3_-as peak is not observed in Fig. 2(b). This is because the intensity of the CH_3_-as peak is weaker than the noise level, considering that the CH_3_-as peak was 16 times weaker than that of CH_3_-ss. Accordingly, *θ*_S_ = 39.2° was used in the analysis. Substituting 39.2° for *θ*_S_, the other parameters to fit the data include *θ*_D_ = 90°, *σ* = 13, *Δθ*_D_ ＝ 0.5, and *Δσ* ＝ 0.2.

*5. Equations for orientation analysis of CH_3_ group*

The intensity of the SFG light, *I_SFG_*(*ω*), is given by

$I_{SFG}\left( \omega\right)=\frac{8\pi^{3}\omega^{2}\sec\beta^{2}}{c_{0}^{3}n_{i}\left( \omega\right)n_{i1}\left( \omega_{1} \right)n_{i2}\left( \omega_{2} \right)}\left| \chi_{SSP}^{(2)} \right|^{2}I\left( \omega_{1} \right)I\left( \omega_{2} \right)$ (2)

where *n*_i_(*ω*_j_) is the refractive index of medium i at frequency *ω*_j_; *ω*, *ω*_1_, and *ω*_2_ are the frequencies of the SFG, VIS, and IR light, respectively; *β* represents the incident and reflection angles of the SFG light; *I*(*ω*_1_) and *I*(*ω*_2_) are the intensities of the incident light of VIS and IR light, respectively; *c*_0_ is the speed of light in vacuum; and *χ*_ssp,S_ is the second-order susceptibility modified by the Fresnel factors. With the ssp polarisation combination, the second-order susceptibility of CH_3_-ss, *χ*_ssp,S_, under the static condition is given by Eq. (3):

$\chi_{ssp, S}^{(2)}\left( \theta_{S} \right)=L_{yy}\left( \omega\right)L_{yy}\left( \omega_{1} \right)L_{zz}\left( \omega_{2} \right)\chi_{yyz}^{(2)}$ (3)

where *L_ij_* (*i*,*j*=x,y,z) is the Fresnel factor. In the case of the CH_3_ group, the vibrational modes of CH_3_ along the molecular axis are assumed to degenerate; thus, the twist angle around the molecular axis (*φ*) can be randomly approximated. In addition, the molecular orientation in the in-plane direction (i.e., azimuthal angle) can also be approximated as random, as shown in Eq. (4).

$\sum_{\chi=0}^{2\pi} \sin n\chi=0,\sum_{\chi=0}^{2\pi} \cos n\chi=0$、$\sum_{\varphi=0}^{2\pi} \sin n\phi=0,\sum_{\varphi=0}^{2\pi} \cos n\phi=0$ (4)

Based on this hypothesis, *χ*^(2)^_yyz_ can be expressed by

$\chi_{yyz}^{(2)}=\frac{1}{2}N_{S}\beta_{ccc}\left[ \left( 1+R \right)\left\langle\cos\theta\right\rangle-\left( 1-R \right)\left\langle\cos^{3} \theta\right\rangle\right]$ (5)

$$R=\frac{\beta_{aac}}{\beta_{ccc}}=\frac{\beta_{bbc}}{\beta_{ccc}}$$

where *β*_ijk_ is hyperpolarisable in the molecular coordinate system (*a*, *b*, *c*) and may be derived from experimental methods using polarised Raman and infrared spectroscopy as well as theoretical calculations, such as the ab initio molecular orbital method. *N*_s_ is the number of molecules at surface in a unit volume. From Eqs. (3) and (5), the second-order susceptibility of CH_3_-ss under static conditions is expressed as a function of the tilt angle, *θ*_S_.

By contrast, the second-order susceptibility of CH_3_-ss, *χ*_ssp,D_, under the rolling contact condition should consider the in-plane anisotropy, which is given by Eq. (6).

$\chi_{ssp, D}^{(2)}\left( \theta_{R},\chi\right)=L_{yy}\left( \omega\right)L_{yy}\left( \omega_{1} \right)L_{zz}\left( \omega_{2} \right)\chi_{yyz}^{(2)}+L_{yy}\left( \omega\right)L_{yy}\left( \omega_{1} \right)L_{xx}\left( \omega_{2} \right)\chi_{yyx}^{(2)}$ (6)

Under dynamic conditions, the anisotropic orientation in the in-plane direction renders the azimuthal angle, *χ*, non-random. Hence, only the twist angle (*φ*) can be approximated as random.

$\sum_{\varphi=0}^{2\pi} \sin n\phi=0,\sum_{\varphi=0}^{2\pi} \cos n\phi=0$ (7)

Based on this hypothesis, *χ*^(2)^_yyz_ and *χ*^(2)^_yyx_ can be expressed by the following.

$$\chi_{yyx}^{(2)}=-\frac{1}{2}N_{S}\left( \beta_{aac}+\beta_{bbc} \right)\sin\theta\cos\chi$$

$+\frac{1}{8}N_{S}\left( \beta_{aac}+\beta_{bbc}-2\beta_{ccc} \right)\sin^{3} \theta\left( \cos\chi-\cos3\chi\right)$ (8a)

$$\chi_{yyz}^{(2)}=\frac{1}{2}N_{S}\left( \beta_{aac}+\beta_{bbc} \right)\cos\theta$$

$-\frac{1}{4}N_{S}\left( \beta_{aac}+\beta_{bbc}-2\beta_{ccc} \right)\left( \cos\theta-\cos^{3} \theta\right)\left( 1-\cos2\chi\right)$ (8b)

In Eqs. (6) and (8), the second-order susceptibility of CH_3_-ss under static contact conditions is expressed as a function of the tilt angle, *θ*_S_, and azimuthal angle, *χ*. The azimuthal angle under the rolling contact condition is regarded to be unoriented but adopts a certain distribution with the median in a certain direction. In this analysis, the distribution of azimuthal angles is assumed to follows a Gaussian distribution:

$N\left( \chi;\mu,\sigma\right)=\frac{1}{\sqrt{2\pi\sigma}}e^{-\frac{1}{2}\left( \frac{\chi-\mu}{\sigma} \right)^{2}}$ (9)

where *μ* is the distribution median, which is assumed in this analysis to be in the frictional direction (forward direction, *χ* = 0°; backward direction *χ* = 180°), and *σ* is the standard deviation. The calculation of the azimuthal angle, *χ*, is distribution proceeds as follows.

- Forward direction:

$\chi_{ssp,D}^{\left( 2 \right)}\left( \theta_{D},\sigma\right)=\frac{\int_{-\pi}^{\pi} N\left( \chi;0,\sigma\right)\times\chi_{ssp, D}^{\left( 2 \right)}\left( \theta_{D},\chi\right)d\chi}{\int_{-\pi}^{\pi} N\left( \chi;0,\sigma\right)}$ (10a)

- Backward direction:

$\chi_{ssp, D}^{\left( 2 \right)}\left( \theta_{D},\sigma\right)=\frac{\int_{0}^{2\pi} N\left( \chi;\pi,\sigma\right)\times\chi_{ssp, D}^{\left( 2 \right)}\left( \theta_{D},\chi\right)d\chi}{\int_{0}^{2\pi} N\left( \chi;0,\sigma\right)}$ (10b)

In the calculation, the azimuthal angle was divided into 36 parts (2*π* /36) and multiplied by the median of χ_ssp,D_ as well as the average of the probable density of the divided parts. The summation of the multiplied values of all 36 parts was treated as χ_ssp,D_.

Upon the first rolling step, assuming that the azimuthal angle has a Gaussian distribution with a median at *χ* = 0°, the second-order susceptibility, χ_ssp,D_, is given by the following.

$$\chi_{ssp, D}^{\left( 2 \right)}\left( \theta_{D},\sigma\right)=\left\{ \chi_{ssp, D}^{\left( 2 \right)}\left( \theta_{D},0 \right)\times\sum_{\chi=-5}^{5} N\left( \chi;0,\sigma\right)+\chi_{ssp, D}^{\left( 2 \right)}\left( \theta_{D},10 \right)\times\sum_{\chi=5}^{15} N\left( \chi;0,\sigma\right)+\ldots\right.$$

$$+ \chi_{ssp, D}^{\left( 2 \right)}\left( \theta_{D},180 \right)\times\left( \sum_{\chi=175}^{180} N\left( \chi;0,\sigma\right)+\sum_{\chi=-180}^{-175} N\left( \chi;0,\sigma\right) \right)+\ldots$$

$\left. + \chi_{ssp, D}^{\left( 2 \right)}\left( \theta_{D},350 \right)\times\sum_{\chi=-15}^{-5} N\left( \chi;0,\sigma\right) \right\}\div\sum_{\chi=-180}^{180} N\left( \chi;0,\sigma\right)$ (11)

Upon the second rolling step, χ_ssp,D_ was calculated with the assumption that the azimuthal angle had a Gaussian distribution with a median at *χ* = 0° because the rolling direction was opposite to that of the first rolling step. Moreover, *θ*_D_ and *σ* may be changed by the rolling motion. The changes in *θ*_D_ and *σ* with rolling motion were assumed to be proportional to the number of rolling steps. The χ_ssp,D_ calculation from the second rolling step included the changes in *θ*_D_ and *σ*; this meant that *Δθ*_D_ (a decreasing change in *θ*_D_ was positive) and *Δσ* (a decreasing change in *σ* was positive) were considered.

$$\chi_{ssp, D}^{\left( 2 \right)}\left( \theta_{D},\sigma\right)=\left\{ \chi_{ssp, D}^{\left( 2 \right)}\left( \theta_{D}-\Delta\theta_{D},0 \right)\times\left( \sum_{\chi=175}^{180} N\left( \chi;180,\sigma-\Delta\sigma\right)+\sum_{\chi=-180}^{-175} N\left( \chi;180,\sigma-\Delta\sigma\right) \right)+\ldots\right.$$

$$+ \chi_{ssp,D}^{\left( 2 \right)}\left( \theta_{D}-\Delta\theta_{D},180 \right)\times\sum_{\chi=-5}^{5} N\left( \chi;180,\sigma-\Delta\sigma\right)+\ldots$$

$\left. + \chi_{ssp, D}^{\left( 2 \right)}\left( \theta_{D}-\Delta\theta_{D},350 \right)\times\sum_{\chi=165}^{175} N\left( \chi;180,\sigma-\Delta\sigma\right) \right\}\div\sum_{\chi=-180}^{180} N\left( \chi;180,\sigma-\Delta\sigma\right)$ (12)

The above calculation was repeated until the ninth rolling step.

$$\chi_{ssp, D}^{\left( 2 \right)}\left( \theta_{D},\sigma\right)=\left\{ \chi_{ssp, D}^{\left( 2 \right)}\left( \theta_{D}-8\Delta\theta_{D},0 \right)\times\sum_{\chi=-5}^{5} N\left( \chi;0,\sigma-8\Delta\sigma\right)+\ldots\right.$$

$$+ \chi_{ssp, D}^{\left( 2 \right)}\left( \theta_{D}-8\Delta\theta_{D},180 \right)\times\left( \sum_{\chi=175}^{180} N\left( \chi;0,\sigma-8\Delta\sigma\right)+\sum_{\chi=-180}^{-175} N\left( \chi;0,\sigma-8\Delta\sigma\right) \right)+\ldots$$

$\left. + \chi_{ssp, D}^{\left( 2 \right)}\left( \theta_{D}-8\Delta\theta_{D},350 \right)\times\sum_{\chi=-15}^{-5} N\left( \chi;0,\sigma-8\Delta\sigma\right) \right\}\div\sum_{\chi=-180}^{180} N\left( \chi;0,\sigma-8\Delta\sigma\right)$ (13)

The intensity ratio, *I*^(2)^_SSP_ (dynamic/static), was calculated from the obtained χ_ssp,S_ and χ_ssp,D_.

$I_{ssp}^{(2)}\left( \mathrm{dynamic}/\mathrm{static} \right)=\frac{I_{ssp,D}^{(2)}\left( \theta_{D},\sigma\right)}{I_{ssp,S}^{(2)}\left( \theta_{S} \right)}$ (14)

The SFG intensity ratio is a function of *θ*_S_, *θ*_D_, *Δθ*_D_, *σ*, and *Δσ*, which are used to fit the experimental data.

*6. Measurement point for SFG and position dependence of SA/HD SFG spectra*

To confirm that the overlapped laser spots of the visible (VIS) and infrared (IR) light at the contact interface, the position dependence of the SFG spectra was measured. Figure 2(a) shows a schematic of the measurement points, which vary in steps of 0.5 ±1 mm from the centre of contact. Figure S6 shows the SFG spectra corresponding to each measurement point in Fig. 2(a). The square frame shown in Fig. S6 corresponds to the spectra shown in Fig. 2(a). In this measurement, the polarisation combination was set to ppp (SFG, VIS, IR) to ensure that the measurement conditions were consistent with those of a previous study ^[4–7]^. In all SFG spectra, a peak of 2940 cm^−1^ attributed to the CH_3_ asymmetric stretching (CH_3_-v_as_) was observed. However, the peak intensity at the contact point (Fig. S6(#3)) was noticeably lower than those at other points (Fig. S6, (#1), (#2), (#4), and (#5)). Previous research on the pressure response of SFG signals as a function of SAMs demonstrated a decrease in the peak intensities at the contact point, leading to the conclusion that pressure caused the interfacial molecular structure disorder at the contact point ^[4–7]^. Hence, in this study, a decrease in the intensity at the contact point indicated that the adsorbed SA film was disordered by the contact pressure. The SFG measurements (presented in this manuscript and explained in the Supplementary Information hereafter) were obtained at the centre of the contact point (Fig. S6 (#3)).

**References**

^1^ Wei, X., Hong, S. C., Zhuang, X., Goto, T. & Shen, Y. R. Nonlinear optical studies of liquid crystal alignment on a rubbed polyvinyl alcohol surface. *Phys. Rev. E Stat. Phys. Plasmas Fluids Relat. Interdiscip. Topics* **62**(4 Pt A), 5160–5172 (2000). [10.1103/physreve.62.5160](https://doi.org/10.1103/physreve.62.5160), Pubmed:[11089064](https://www.ncbi.nlm.nih.gov/pubmed/11089064)

^2^ Simpson, G. J. & Rowlen, K. L. An SHG magic angle: dependence of second harmonic generation orientation measurements on the width of the orientation distribution. *Journal of American Chemical Society* **121**(11), 2635–2636 (1999). [10.1021/ja983683f](https://doi.org/10.1021/ja983683f)

^3^ Wang, J., Paszti, Z., Even, M. A. & Chen, Z. Measuring polymer surface ordering differences in air and water by sum frequency generation vibrational spectroscopy. *Journal of American Chemical Society* **124**(24), 7016–7023 (2002). [10.1021/ja012387r](https://doi.org/10.1021/ja012387r), Pubmed:[12059225](https://www.ncbi.nlm.nih.gov/pubmed/12059225)

^4^ Beattie, D. A., Haydock, S. & Bain, C. D. A comparative study of confined organic monolayers by Raman scattering and sum-frequency spectroscopy. *Vib. Spectrosc.* **24**(1), 109–123 (2000). [10.1016/S0924-2031(00)00084-9](https://doi.org/10.1016/S0924-2031(00)00084-9)

^5^ Berg, O. & Klenerman, D. Vibrational spectroscopy of mechanically compressed monolayers. *J. Am. Chem. Soc.* **125**(18), 5493–5500 (2003). [10.1021/ja027128w](https://doi.org/10.1021/ja027128w), Pubmed:[12720464](https://www.ncbi.nlm.nih.gov/pubmed/12720464)

^6^ Meltzer, C., Jä Ger, C. M., Clark, T., Zahn, D., Braunschweig, B. & Peukert, W. Indentation and self-healing mechanisms of a self-assembled monolayer--a combined experimental and modeling study. *J. Am. Chem. Soc.* **136**(30), 10718–10727 (2014). [10.1021/ja5048076](https://doi.org/10.1021/ja5048076), Pubmed:[25054614](https://www.ncbi.nlm.nih.gov/pubmed/25054614)

^7^ Ghalgaoui, A. *et al.* Monolayer study by VSFS: In situ response to compression and shear in a contact. *Langmuir* **30**(11), 3075–3085 (2014). [10.1021/la4042474](https://doi.org/10.1021/la4042474), Pubmed:[24547702](https://www.ncbi.nlm.nih.gov/pubmed/24547702)


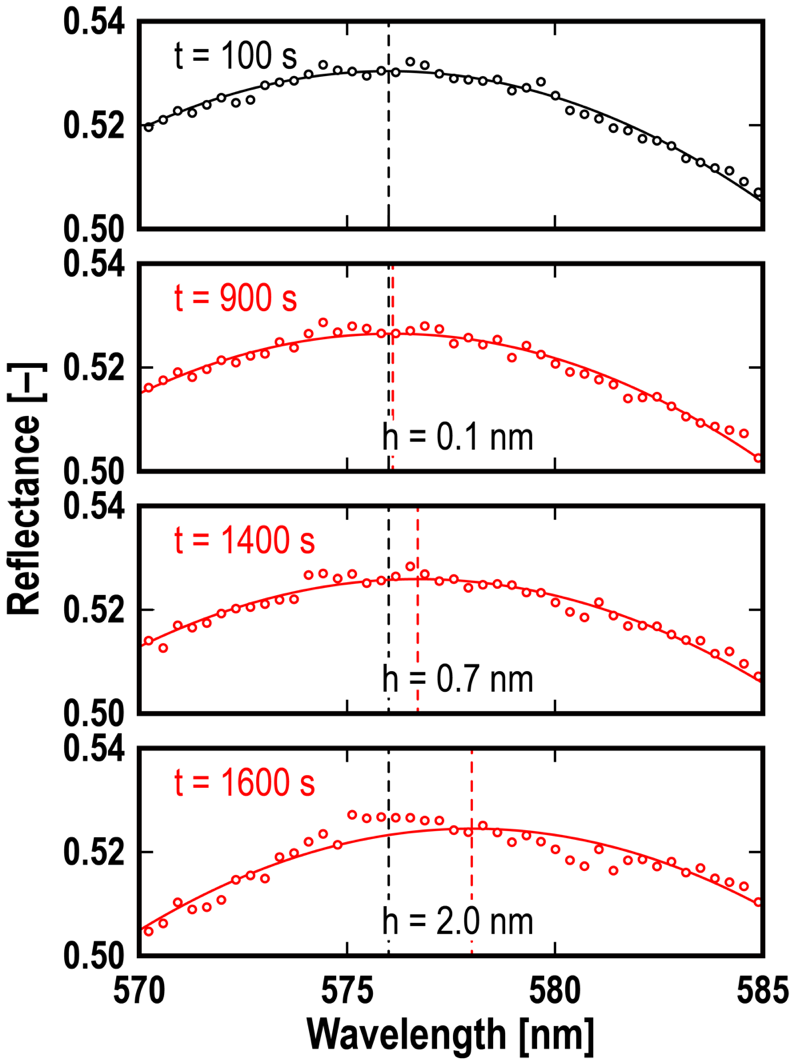


**Fig. S1** Effect of the lubricant film thickness on the peak wavelength of the reflectance as measured by UTFI. Top row: spectrum of SA/HD at *t* = 100 s; Second row: spectrum of SA/HD at *t* = 900 s, which was used to determine the film thickness, *h* = 0.1 nm, at t = 900 s; Third row: spectrum of SA/HD at *t* = 1400 s, which was used to determine the film thickness, *h* = 0.7 nm, at t = 1400 s; Bottom row: spectrum of SA/HD at *t* = 1600 s, which was used to determine the film thickness, *h* = 2.0 nm, at t = 1600 s; black plots: spectrum measured at *t* = 100 s; red plots: each spectrum was measured at *t* = 900 s, *t* = 1400 s, and *t* = 1600 s; solid line: fitting curves for measured plots; black dashed line: peak wavelength of fitting curve at *t* = 100 s; red dashed lines: peak wavelength of each fitting curve at *t* = 900 s, *t* = 1400 s, and *t* = 1600 s.


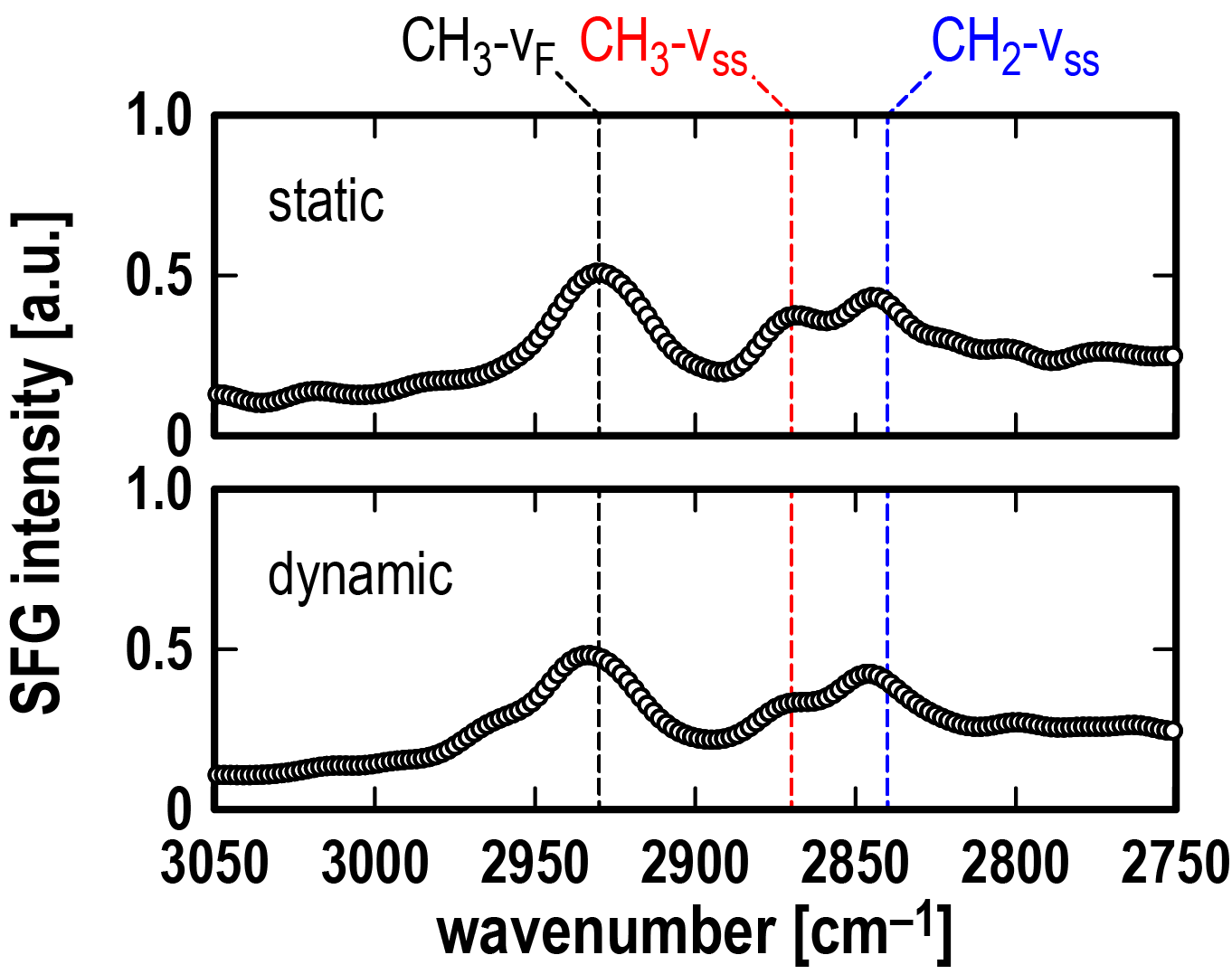


**Fig. S2** SFG spectra of SA-d_35_/HD under static and dynamic contact conditions.


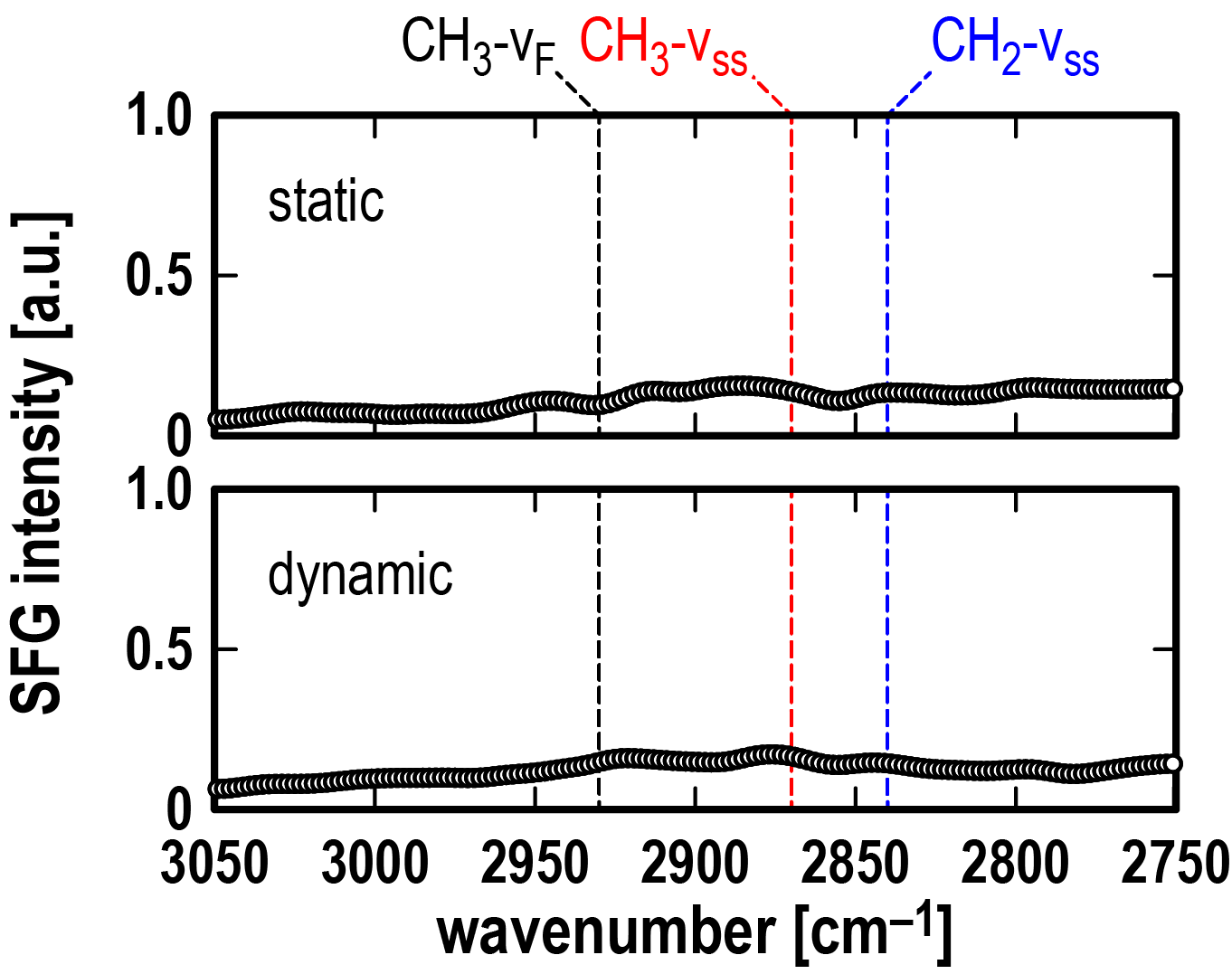


**Fig. S3** SFG spectra of HD under static and dynamic contact conditions.


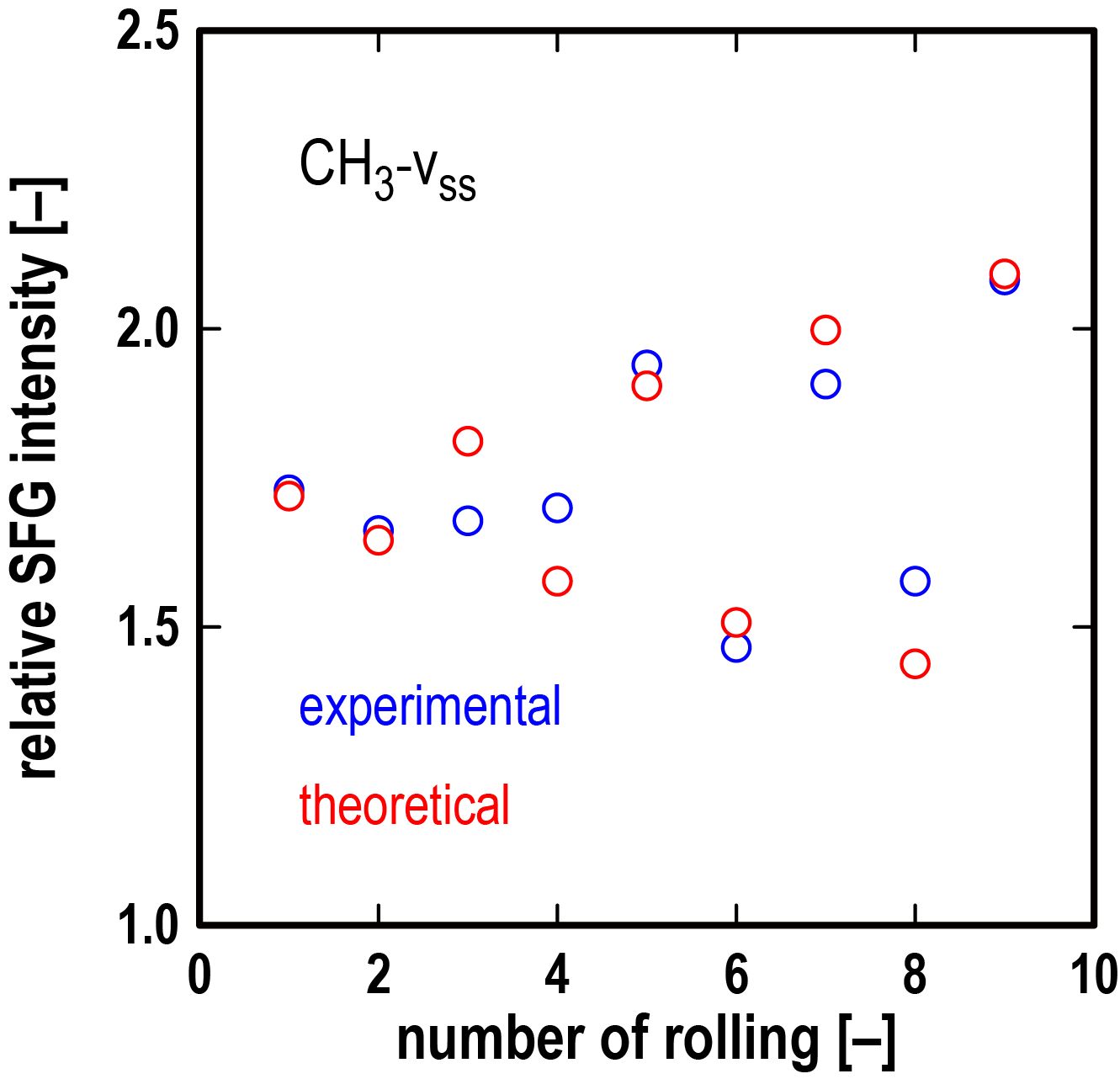


**Fig. S4** Comparison of the SFG intensity ratio between the experimental and theoretical results that considers changes in the tilt angle, *θ*, and azimuthal angle, *χ*.


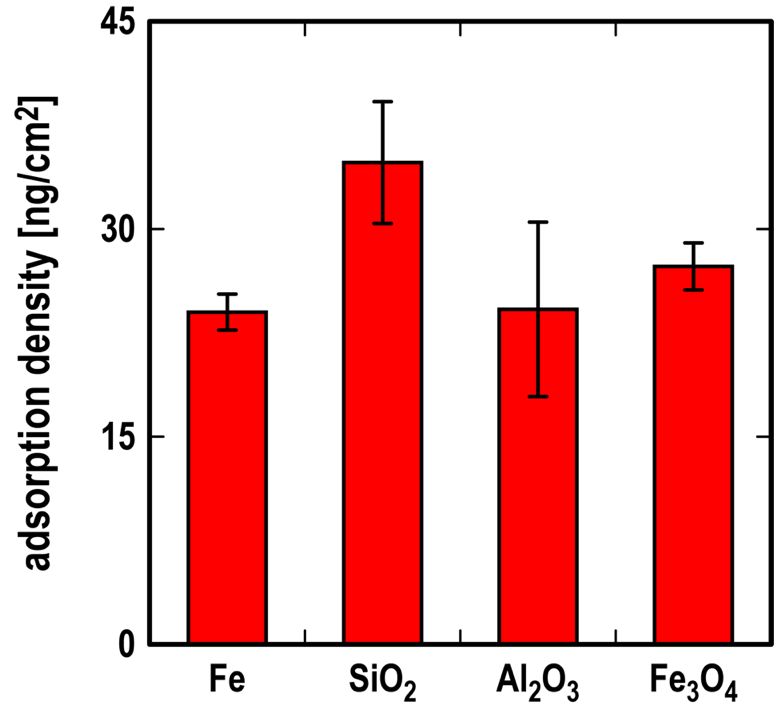


**Fig. S5** Density of adsorbed SA/HD molecules on the Fe, SiO_2_, Al_2_O_3_, and Fe_3_O_4_ QCM sensors.


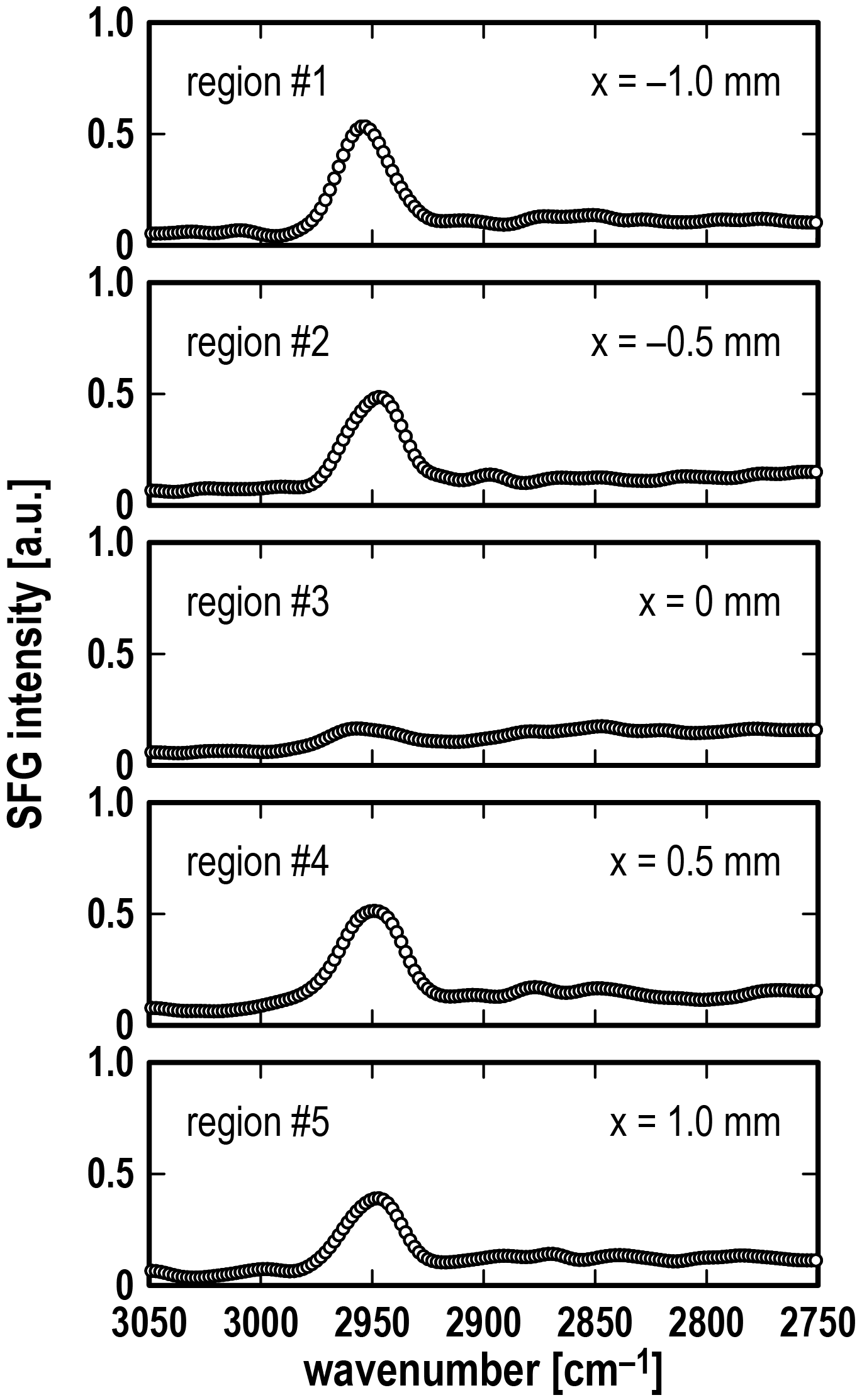


**Fig. S6** SFG spectra at various measurement positions (shown in the inset in Fig. 2a): (#1) 1.0 mm to the left of centre; (#2) 0.5 mm to the left of centre; (#3) at the centre (the measurement point of the experiment in the study); (#4) 0.5 mm to the right of centre, and (#5) 1.0 mm to the right of centre.

**Table S1** Influence of centre wavelength of second-order approximation on *R*^2^.

**Table S2** Dataset of fitting parameters for time-dependent SFG measurement.
